# Identification of Predictive Markers for Response to Neoadjuvant Chemoradiation in Rectal Carcinomas by Proteomic Isotope Coded Protein Label (ICPL) Analysis

**DOI:** 10.3390/ijms17020209

**Published:** 2016-02-04

**Authors:** Roland S. Croner, Müzeyyen Sevim, Metodi V. Metodiev, Peter Jo, Michael Ghadimi, Vera Schellerer, Maximillian Brunner, Carol Geppert, Tilman Rau, Michael Stürzl, Elisabeth Naschberger, Klaus E. Matzel, Werner Hohenberger, Friedrich Lottspeich, Josef Kellermann

**Affiliations:** 1 Department of Surgery, University Hospital Erlangen, Erlangen 91054, Germany; vera.schellerer@uk-erlangen.de (V.S.); maximilian.brunner@uk-erlangen.de (M.B.); klaus.matzel@uk-erlangen.de (K.E.M.); werner.hohenberger@uk-erlangen.de (W.H.); 2 Max-Planck-Institute of Biochemistry, Martinsried 82152, Germany; sevim@gmx.net (M.S.); lottspei@biochem.mpg.de (F.L.); kellerma@biochem.mpg.de (J.K.); 3 School of Biological Sciences/Proteomics Unit, University of Essex; Wivenhoe Park, Colchester, Essex CO4 3SQ, UK; mmetod@essex.ac.uk; 4 Department of Surgery, University Hospital Göttingen, Göttingen 37075, Germany; jo.peter@chirurgie-goettingen.de (P.J.); mghadimi@chirurgie-goettingen.de (M.G.); 5 Department of Pathology, University Hospital Erlangen, Erlangen 91054, Germany; carol.geppert@uk-erlangen.de (C.G.); tilman.rau@pathology.unibe.ch (T.R.); 6 Division of Molecular and Experimental Surgery, University Hospital Erlangen, Erlangen 91054, Germany; michael.stuerzl@uk-erlangen.de (M.S.); elisabeth.naschberger@uk-erlangen.de (E.N.)

**Keywords:** rectal cancer, chemoradiation, proteomic, ICPL

## Abstract

Neoadjuvant chemoradiation (nCRT) is an established procedure in stage union internationale contre le cancer (UICC) II/III rectal carcinomas. Around 53% of the tumours present with good tumor regression after nCRT, and 8%–15% are complete responders. Reliable selection markers would allow the identification of poor or non-responders prior to therapy. Tumor biopsies were harvested from 20 patients with rectal carcinomas, and stored in liquid nitrogen prior to therapy after obtaining patients’ informed consent (Erlangen-No.3784). Patients received standardized nCRT with 5-Fluoruracil (nCRT I) or 5-Fluoruracil ± Oxaliplatin (nCRT II) according to the CAO/ARO/AIO-04 protocol. After surgery, regression grading (Dworak) of the tumors was performed during histopathological examination of the specimens. Tumors were classified as poor (Dworak 1 + 2) or good (Dworak 3 + 4) responders. Laser capture microdissection (LCM) for tumor enrichment was performed on preoperative biopsies. Differences in expressed proteins between poor and good responders to nCRT I and II were identified by proteomic analysis (Isotope Coded Protein Label, ICPL™) and selected markers were validated by immunohistochemistry. Tumors of 10 patients were classified as histopathologically poor (Dworak 1 or 2) and the other 10 tumor samples as histopathologically good (Dworak 3 or 4) responders to nCRT after surgery. Sufficient material in good quality was harvested for ICPL analysis by LCM from all biopsies. We identified 140 differentially regulated proteins regarding the selection criteria and the response to nCRT. Fourteen of these proteins were synchronously up-regulated at least 1.5-fold after nCRT I or nCRT II (e.g., FLNB, TKT, PKM2, SERINB1, IGHG2). Thirty-five proteins showed a complete reciprocal regulation (up or down) after nCRT I or nCRT II and the rest was regulated either according to nCRT I or II. The protein expression of regulated proteins such as PLEC1, TKT, HADHA and TAGLN was validated successfully by immunohistochemistry. ICPL is a valid method to identify differentially expressed proteins in rectal carcinoma tissue between poor *vs.* good responders to nCRT. The identified protein markers may act as selection criteria for nCRT in the future, but our preliminary findings must be reproduced and validated in a prospective cohort.

## 1. Introduction

Neoadjuvant chemoradiation (nCRT) is an established procedure for locally advanced rectal carcinomas stage union internationale contre le cancer (UICC) II and lymph node positive rectal carcinomas stage UICC III [1,2,3,4,5]. Local tumor control is improved by nCRT. The percentage of sphincter preservation rate is increased and local recurrence rates after complete surgical removal of the tumor (R0) is decreased [4,6]. No improvement on overall patients’ survival is noted after nCRT [4]. The response to nCRT varies considerably with the patient cohorts. Applying the histopathological regression grading described by Dworak, poor regression was observed in 15%, intermediate regression in 70% and complete regression in 15% of the carcinomas [7]. The regression rate seems to improve prognosis significantly and there may be a correlation between tumor response, distant metastasis and patients’ survival [8]. However, in 6% of complete tumor response to nCRT (ypT0) lymph node metastases can be detected in the resected specimens. Lymph node metastases are considered as crucial prognostic indicators [8].

Neoadjuvant therapy comes with costs, not only is it associated with toxic side effects but it also represents an economic burden. Because the therapeutic effect of nCRT is variable and around 15% of the treated patients do not experience a therapeutic benefit at all, a case selection prior to therapy is desirable [9]. But currently no predictive indicators for nCRT response exist for routine clinical use. Today selection for nCRT is based on imaging techniques. These imaging techniques have limitations in detecting tumor-affected lymph nodes. Molecular markers of the tumor would be ideal to select patients, because they can be analysed prior to therapy in endoscopically harvested tissues. As of today no such markers exist.

To identify molecular markers predictive for nCRT therapy response, we analyzed the proteome of laser captured micro dissected rectal carcinoma biopsies prior to nCRT with isotope coded protein label (ICPL), a recent quantitative technique for protein analysis [10,11]. These results were postoperatively correlated with histopathological tumor response grade (Dworak) of the resected specimens.

## 2. Results

### 2.1. Tumor Samples and Laser Capture Microdissection

We harvested two tumor biopsies from rectal carcinomas from each patient prior to nCRT. One was harvested in liquid nitrogen and stored at −80 °C for proteome analysis, the second one underwent formalin fixation and paraffin embedding procedure (FFPE) fixation for immunohistochemistry. No complications occurred during biopsy sampling. Fresh frozen samples were cut in 8–10 µm slices and prepared for Laser capture microdissection (LCM) as described. A total of 100 mm^2^ pure tumor tissue was collected by LCM. There was sufficient tumor material within a 5 mm tumor biopsy to harvest this amount of carcinoma. From 100 mm^2^ of tumor tissue an amount of 20 µg total protein could be isolated for ICPL.

### 2.2. Proteome Analysis by Isotope Coded Protein Label (ICPL)

The proteome analysis of the 2 × 24 OFFGEL-fraction and the non-fractionated samples (50 samples in total) was performed via ICPL-ESI*Quant* Software. Proteins were referred as quantified and identified with at least two multiplets per protein and a unique peptide per protein. A protein was identified as a single protein species in neighbouring OFFGEL-fractions, whereas, a protein was identified as a separate protein species or isoform in several non side-by-side OFFGEL-fractions. In total 3222 protein species were detected in all 24 fractions of the first OFFGEL-analysis (645 unique protein species). However, because many of the proteins have been found in several fractions, this number was reduced to 131. In the second technical replicate, 3734 protein species were detected in total in all 24 fractions of the OFFGEL-analysis (734 unique protein species). Likewise proteins appearing in several fractions were removed, with 146 proteins left over. Eight-two proteins were identical in both technical replicates. In the first analysis of the not-fractionated samples, from in total 291 detected protein species, 62 unique proteins were identified. The second analysis of the non-fractionated samples could not be included in the evaluation, because that generated by the LC-MS/MS spectra did not permit reliable statements. A repeat of the experiment could not be performed due to insufficient sample amount. Forty-two of the identified proteins were found in all analyses (fractionated and non-fractionated). The identified proteins were classified as differentially expressed with a regulation value of 1.5 ≥ 1 ≥ 0.66, with at least two quadruplets per protein and a unique peptide per protein (CV ≤ 30%). The analysis for nCRT I and nCRT II was carried out separately because of the different chemotherapy regimens added to radiation therapy. Thus, in the data set of nCRT I, 201 proteins (non-redundant) were identified in 2 × 24 OFFGEL fractions and in the non-fractioned sample and the corresponding protein IDs are allocated from the IPI database; of these, 140 proteins could meet the described regulation value. Out of these 140 differentially regulated proteins, 79 proteins are downregulated in the proteome of poor/moderate responses of nCRT I and 61 proteins were upregulated. In the data set of nCRT II, and 201 proteins (non-redundant) were identified in 2 × 24 OFFGEL fractions and in the non-fractioned sample, and the corresponding protein IDs are allocated from the IPI database. Of these, 114 proteins met the described regulation values. Out of these 114 differentially regulated proteins, 91 proteins are downregulated in the proteome of poor/moderate responses of nCRT 1 and 23 proteins were upregulated.

Fourteen of these proteins showed a synchronous regulation after nCRT I and 2: A high expression of FLNB Isoform 1 of Filamin-B, Transketolase, PKM2 Isoform M2 of Pyruvate kinase isozymes M1/M2 and SERPINB1 Leukocyte elastase inhibitor and a low expression of IGHG2, Putative uncharacterized protein DKFZp686C15213 was particularly predictive for nCRT without any preference to the added chemotherapy throughout the applied RCT regiments. Thirty-five proteins were completely reciprocally regulated in response to nCRT I *vs.* nCRT II. Transgelin, LOC440786 Ig kappa chain V–II region TEW and kappa light chain variable region (Fragment) were highly expressed in good responders to nCRT II and expressed at very low levels in good responders to nCRT I. But heterogeneous nuclear ribonucleoprotein F, Adenine phosphoribosyltransferase (APRT) or Tubulin α-1C chain (TUBA1C) were highly expressed in response to nCRT I and expressed at very low levels in response to nCRT II. Eighty-nine proteins were regulated either in response to nCRT I or II. Of these proteins thirty eight were upregulated and 20 down-regulated in good response to nCRT I. Peroxiredoxin-4 (PRDX4), Tubulin α-1A chain (TUBA1A) and Fatty acid-binding protein, epidermal (FABP5; FABP5L7) were highly expressed and Isoform 1 of Fibrinogen α chain precursor (FGA), Isoform 1 of Caldesmon (CALD1) or Isoform Gamma-A of Fibrinogen gamma chain precursor (FGG) were very low expressed in response to nCRT I. Twenty-one proteins were up and 10 proteins were down regulated in response to nCRT II. Of these proteins Actin, α skeletal muscle (ACTA1), Actin, α cardiac muscle 1 (ACTC1) and filamin A, α isoform 1 (FLNA) were extremely highly expressed and Histone H1.5 (HIST1H1B) and 60S ribosomal protein L1i3a (RPL13A) were expressed at very low levels in response to nCRT II. A detailed list of differentially regulated proteins is provided in Table 1.

**Table 1:** Differentially regulated proteins identified by isotope coded protein label (isotope coded protein label (ICPL)) between poor (Dworak 1 + 2) and good (Dworak 3 + 4) responders to neoadjuvant chemoradiation (nCRT) I or II; n.r.: not regulated regarding the selection criteria (regulation value 1.5 ≥ 1 ≥ 0.66 and CV ≤ 30%); nCRT I: 50.4 Gy + 5-FU; nCRT II: 50.4 Gy + 5-FU/Oxaliplatin.

Accession Number	Protein Name	Protein Expression ICPL in nCRT I (Dworak 1 + 2/Dworak 3 + 4)	Protein Expression ICPL in nCRT II (Dworak 1+2/Dworak 3 + 4)
Synchronously regulated proteins in nCRT I and II			
IPI00006663	ALDH2 Aldehyde dehydrogenase, mitochondrial precursor	0.53	0.53
IPI00010290	FABP1 FABP1 protein (Fragment)	0.54	0.33
IPI00013847	UQCRC1 Cytochrome b-c1 complex subunit 1, mitochondrial precursor	0.63	0.52
IPI00014898	PLEC1 Isoform 1 of Plectin-1	0.48	0.43
IPI00026185	CAPZB Isoform 1 of F-actin-capping protein subunit beta	0.64	0.58
IPI00027444	SERPINB1 Leukocyte elastase inhibitor	0.30	0.49
IPI00216256	WDR1 Isoform 2 of WD repeat-containing protein 1	0.40	0.56
IPI00289334	FLNB Isoform 1 of Filamin-B	0.36	0.39
IPI00298860	LTF Growth-inhibiting protein 12	0.56	0.41
IPI00337335	MYH14 Isoform 1 of Myosin-14	0.41	0.57
IPI00426051	IGHG2, Putative uncharacterized protein DKFZp686C15213	2.36	2.35
IPI00479186	PKM2 Isoform M2 of Pyruvate kinase isozymes M1/M2	0.43	0.43
IPI00643920	TKT Transketolase	0.38	0.47
IPI00793199	ANXA4 annexin IV	0.41	0.58
Up regulated proteins in good responders (Dworak 3 + 4) nCRT I			
IPI00000105	MVP Major vault protein	0.48	n.r.
IPI00000874	PRDX1 Peroxiredoxin-1	0.59	n.r.
IPI00004657	HLA-B major histocompatibility complex, class I, B	0.45	n.r.
IPI00007750	TUBA4A Tubulin α-4A chain	0.37	n.r.
IPI00007752	TUBB2C Tubulin beta-2C chain	0.40	n.r.
IPI00007797	FABP5;FABP5L7 Fatty acid-binding protein, epidermal	0.31	n.r.
IPI00008274	CAP1 Adenylyl cyclase-associated protein	0.55	n.r.
IPI00010133	CORO1A Coronin-1A	0.57	n.r.
IPI00010154	GDI1 Rab GDP dissociation inhibitor α	0.53	n.r.
IPI00011654	TUBB Tubulin beta chain	0.51	n.r.
IPI00011937	PRDX4 Peroxiredoxin-4	0.11	n.r.
IPI00013683	TUBB3 Tubulin beta-3 chain	0.50	n.r.
IPI00013881	HNRPH1 Heterogeneous nuclear ribonucleoprotein H	0.59	n.r.
IPI00013890	SFN Isoform 1 of 14-3-3 protein sigma	0.44	n.r.
IPI00024095	ANXA3 Annexin A3	0.51	n.r.
IPI00025252	PDIA3 Protein disulfide-isomerase A3 precursor	0.49	n.r.
IPI00025874	RPN1 Dolichyl-diphosphooligosaccharide--protein glycosyltransferase 67 kDa subunit precursor	0.50	n.r.
IPI00027463	S100A6 Protein S100-A6	0.49	n.r.
IPI00028931	DSG2 Desmoglein-2 precursor	0.49	n.r.
IPI00031461	GDI2 Rab GDP dissociation inhibitor beta	0.56	n.r.
IPI00169383	PGK1 Phosphoglycerate kinase 1	0.42	n.r.
IPI00171903	HNRPM Isoform 1 of Heterogeneous nuclear ribonucleoprotein M	0.65	n.r.
IPI00180675	TUBA1A Tubulin α-1A chain	0.31	n.r.
IPI00216049	HNRPK Isoform 1 of Heterogeneous nuclear ribonucleoprotein K	0.50	n.r.
IPI00218782	CAPZB Capping protein	0.64	n.r.
IPI00218852	VIL1 Villin-1	0.50	n.r.
IPI00219153	RPL22 60S ribosomal protein L22	0.53	n.r.
IPI00220644	PKM2 Isoform M1 of Pyruvate kinase isozymes M1/M2	0.53	n.r.
IPI00220739	PGRMC1 Membrane-associated progesterone receptor component 1	0.56	n.r.
IPI00297779	CCT2 T-complex protein 1 subunit beta	0.46	n.r.
IPI00299000	PA2G4 Proliferation-associated protein 2G4	0.46	n.r.
IPI00401264	TXNDC4 Thioredoxin domain-containing protein 4 precursor	0.48	n.r.
IPI00410693	SERBP1 Isoform 1 of Plasminogen activator inhibitor 1 RNA-binding protein	0.51	n.r.
IPI00419585	PPIA;PPIAL3;LOC654188 Peptidyl-prolyl cis-trans isomerase A	0.59	n.r.
IPI00419880	RPS3A 40S ribosomal protein S3a	0.39	n.r.
IPI00465431	LGALS3 Galectin-3	0.46	n.r.
IPI00465439	ALDOA Fructose-bisphosphate aldolase A	0.47	n.r.
IPI00472855	HLA-A HLA class I histocompatibility antigen, A-30 α chain precursor	0.45	n.r.
Down regulated proteins in good responders (Dworak 3 + 4) nCRT I			
IPI00010790	BGN Biglycan precursor	2.46	n.r.
IPI00014516	CALD1 Isoform 1 of Caldesmon	3.16	n.r.
IPI00020986	LUM Lumican precursor	2.22	n.r.
IPI00021885	FGA Isoform 1 of Fibrinogen α chain precursor	4.12	n.r.
IPI00021891	FGG Isoform Gamma-B of Fibrinogen gamma chain precursor	3.04	n.r.
IPI00022391	APCS Serum amyloid P-component precursor	2.41	n.r.
IPI00022395	C9 Complement component C9 precursor	1.74	n.r.
IPI00022418	FN1 Isoform 1 of Fibronectin precursor	2.26	n.r.
IPI00029717	FGA Isoform 2 of Fibrinogen α chain precursor	1.90	n.r.
IPI00031008	TNC Isoform 1 of Tenascin precursor	1.89	n.r.
IPI00168728	IGHM FLJ00385 protein (Fragment)	1.82	n.r.
IPI00215983	CA1 Carbonic anhydrase 1	1.79	n.r.
IPI00216134	TPM1 tropomyosin 1 α chain isoform 7	3.04	n.r.
IPI00218695	CALD1 Isoform 3 of Caldesmon	2.55	n.r.
IPI00219713	FGG Isoform Gamma-A of Fibrinogen gamma chain precursor	3.05	n.r.
IPI00298497	FGB Fibrinogen beta chain precursor	2.64	n.r.
IPI00399007	IGHG2 Putative uncharacterized protein DKFZp686I04196 (Fragment)	1.83	n.r.
IPI00472961	IGKC IGKC protein	2.08	n.r.
IPI00550640	IGHG4 IGHG4 protein	2.30	n.r.
IPI00553153	ATPIF1 Putative uncharacterized protein DKFZp564G0422	1.86	n.r.
Up regulated proteins in good responders (Dworak 3 + 4) nCRT II			
IPI00001539	ACAA2 3-ketoacyl-CoA thiolase, mitochondrial	n.r.	0.48
IPI00003269	Beta-actin-like protein 2_DKFZp686D0972 hypothetical protein LOC345651	n.r.	0.52
IPI00008603	ACTA2 Actin, aortic smooth muscle	n.r.	0.58
IPI00011107	IDH2 Isocitrate dehydrogenase [NADP], mitochondrial precursor	n.r.	0.23
IPI00013508	ACTN1 α-actinin-1	n.r.	0.52
IPI00019502	MYH9 Myosin-9	n.r.	0.40
IPI00021428	ACTA1 Actin, α skeletal muscle	n.r.	0.19
IPI00021439	ACTB Actin, cytoplasmic 1	n.r.	0.57
IPI00021440	Actin, cytoplasmic 2	n.r.	0.45
IPI00023006	ACTC1 Actin, α cardiac muscle 1	n.r.	0.29
IPI00024145	VDAC2 Voltage-dependent anion-selective channel protein 2	n.r.	0.46
IPI00024870	MYH11 smooth muscle myosin heavy chain 11 isoform SM2A	n.r.	0.50
IPI00024919	PRDX3 Thioredoxin-dependent peroxide reductase, mitochondrial precursor	n.r.	0.53
IPI00031522	HADHA Trifunctional enzyme subunit α, mitochondrial precursor	n.r.	0.38
IPI00103467	ALDH1B1 Aldehyde dehydrogenase X, mitochondrial precursor	n.r.	0.27
IPI00216308	VDAC1 Voltage-dependent anion-selective channel prote	n.r.	0.45
IPI00217975	LMNB1 Lamin-B1	n.r.	0.47
IPI00291006	MDH2 Malate dehydrogenase, mitochondrial precursor	n.r.	0.46
IPI00302592	FLNA filamin A, α isoform 1	n.r.	0.22
IPI00418169	ANXA2 annexin A2 isoform 1	n.r.	0.50
IPI00555733	Actin-like protein (Fragment)	n.r.	0.61
Down regulated proteins in good responders (Dworak 3 + 4) nCRT II			
IPI00002535	FKBP2 FK506-binding protein 2 precursor_Peptidyl-prolyl cis-trans isomerase FKBP2	n.r.	1.70
IPI00014263	EIF4H;LOC653994 Isoform Long of Eukaryotic translation initiation factor 4H	n.r.	1.85
IPI00021841	APOA1 Apolipoprotein A-I precursor	n.r.	1.78
IPI00032313	S100A4 Protein S100-A4	n.r.	0.23
IPI00102821	MGC29506 hypothetical protein LOC51237	n.r.	2.58
IPI00217468	HIST1H1B Histone H1.5	n.r.	2.16
IPI00304612	RPL13A 60S ribosomal protein L13a	n.r.	1.83
IPI00412714	SERBP1 Isoform 4 of Plasminogen activator inhibitor 1 RNA-binding protein	n.r.	1.68.
IPI00470498	SERBP1 Isoform 3 of Plasminogen activator inhibitor 1 RNA-binding protein	n.r.	1.71
IPI00479997	STMN1 Stathmin	n.r.	1.65
Reciprocal regulated proteins in nCRT I and II			
IPI00000230	TPM1 tropomyosin 1 α chain isoform 2	2.58	0.47
IPI00003881	HNRPF Heterogeneous nuclear ribonucleoprotein F	0.31	2.26
IPI00003949	UBE2N Ubiquitin-conjugating enzyme E2 N	0.52	1.51
IPI00004573	PIGR Polymeric immunoglobulin receptor precursor	0.56	1.78
IPI00008176	SHROOM4 Isoform 1 of Protein Shroom4	2.26	0.53
IPI00022792	MFAP4 Microfibril-associated glycoprotein 4 precursor	4.81	0.09
IPI00096066	SUCLG2 Succinyl-CoA ligase (GDP-forming) beta-chain, mitochondrial precursor	2.13	0.46
IPI00010779	TPM4 Isoform 1 of Tropomyosin α-4 chain	2.44	0.30
IPI00016801	GLUD1 Glutamate dehydrogenase 1, mitochondrial precursor	1.18	0.50
IPI00018853	TPM1 Tropomyosin isoform	1.87	0.54
IPI00020501	MYH11 Myosin-11	3.09	0.43
IPI00218820	TPM2 Isoform 3 of Tropomyosin beta chain	2.09	0.52
IPI00218693	APRT Adenine phosphoribosyltransferase	0.53	2.02
IPI00219757	GSTP1 Glutathione S-transferase P	043	1.85
IPI00025512	HSPB1 Heat shock protein beta-1	1.55	0.41
IPI00027350	PRDX2 Peroxiredoxin-2	2.18	0.49
IPI00183968	TPM3 tropomyosin 3 isoform 1	2.00	0.30
IPI00216135	TPM1 Isoform 3 of Tropomyosin α-1 chain	2.28	0.51
IPI00216138	TAGLN Transgelin	4.03	0.28
IPI00218319	TPM3 Isoform 2 of Tropomyosin α-3 chain	2.44	0.56
IPI00218343	TUBA1C Tubulin α-1C chain	0.36	2.10
IPI00220362	HSPE1 10 kDa heat shock protein, mitochondrial	1.78	0.49
IPI00220709	TPM2 Isoform 2 of Tropomyosin beta chain	2.52	0.51
IPI00299547	LCN2 Neutrophil gelatinase-associated lipocalin precursor	0.46	1.90
IPI00333771	CALD1 Isoform 5 of Caldesmon	3.16	0.55
IPI00335168	MYL6 Isoform Non-muscle of Myosin light polypeptide 6	2.18	0.49
IPI00382606	F7 Factor VII active site mutant immunoconjugate	2.55	0.53
IPI00549291	IGHM IGHM protein	2.29	0.24
IPI00604537	TPM1 tropomyosin 1 α chain isoform 3	2.55	0.47
IPI00736885	LOC440786 Ig kappa chain V-II region TEW	3.38	0.34
IPI00743194	Kappa light chain variable region (Fragment)	3.38	0.34
IPI00746963	IGKC IGKC protein	2.03	0.43
IPI00815926	IGHG1 IGHG1 protein	2.16	0.47
IPI00843757	TPM2 Uncharacterized protein TPM2 (Fragment)	1.96	0.19
IPI00876888	IGHV4-31 immunoglobulin heavy variable 4-31- cDNA FLJ78387	2.37	0.20

### 2.3. Immunohistochemistry

**Figure 1:**
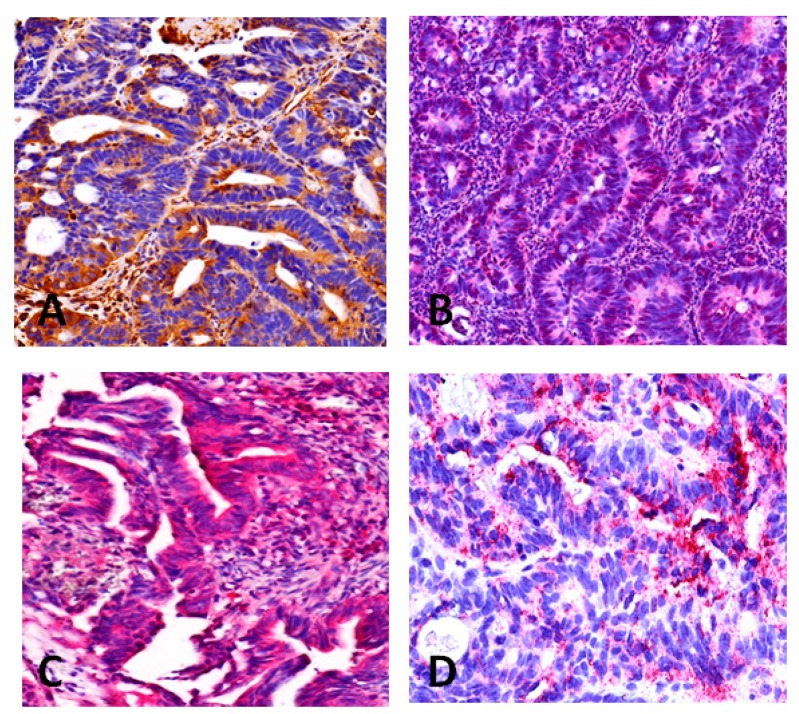
Immunohistochemical validation of protein expression in colorectal carcinoma biopsy tissue identified by isotope coded protein label (ICPL); (**A**) Plectin-1; (**B**) Transketolase; (**C**) Transgelin; (**D**) HADHA Trifunctional enzyme subunit α, mitochondrial precursor.

The validation of protein expression from the randomly selected markers: Plectin-1 (PLEC1), Transketolase (TKT), HADHA Trifunctional enzyme subunit α, mitochondrial precursor and Transgelin (TAGLN) by immunohistochemistry was successful in all cases. The proteins showed clear expression within the tumor cells either in the nucleus or cytoplasm (Figure 1).

## 3. Discussion

We identified a panel of predictive markers for nCRT response in rectal carcinomas with ICPL. ICPL was recently described as powerful tool to identify thousands of proteins in extracts of body fluids or tissue. The technique is characterized by a highly accurate and reproducible quantification method for proteins [10,11]. ICPL is especially useful to identify regulated proteins in different samples. It is an innovative technology, which showed its value for marker screening in human tissue samples in our study. It was possible to validate randomly selected proteins by immunohistochemistry of tumor biopsies. This is a further indicator for the reliability of the identified proteins. One crucial point is the amount of tissue needed for proteome analysis. We harvested one tumor biopsy for proteomics which is generally also needed for histological diagnosis under clinical routine conditions. No patient suffered from any complication during sample harvesting. From our biopsies sufficient material was accumulated after LCM to perform ICPL. Thus ICPL not only provides reliable results but is also a valuable tool for proteome studies of human specimens which are harvested under clinical routine conditions.

The patients who underwent nCRT were all included in a clinical trial (CAO/ARO/AIO-04) [12]. This is important because not only standardized tissue sampling and preparation but also standardized patient care and treatment increases the value of molecular predictive marker evaluation. In our cohort two different types of chemotherapy were added to radiation. It is not surprising that several proteins were regulated only in relation to nCRT I or II. These markers may reflect the response to the different chemotherapy regiments. However 14 markers were uniformly regulated in response to radiation. Chemotherapy regiments added to nCRT change more frequently than radiation itself. Therefore these proteins and especially the very highly expressed ones, which indicate good radiation response, may be the most valuable for further validation in the future, because a highly expressed marker such as a stable protein can easily be identified e.g., by immunohistochemistry in a routinely sampled tumor biopsy under clinical conditions. PKM2, which was one of these markers, was recently described as highly expressed in colorectal cancer and correlated with later stage, lymph node metastases and oxaliplatin metabolism [13,14]. It is a cytosolic enzyme involved in nucleic acid, phospholipid- and amino acid synthesis, which provides critical cell building materials for highly proliferating cells, such as tumor cells. A high expression of Transketolase was already described in colorectal and urothelial cancer and was associated with poor prognosis. In breast cancer Transketolase was identified as a potential target against tumor growth [15]. It is involved in the non-oxidative part of the pentose phosphate pathway which is important during cell metabolism. A decrease in Transketolase expression levels in tumor cells was assumed to delay tumor growth. These findings are indicators that several of the markers identified in this study are related to cancer.

We classified our patients for good and poor nCRT response referring to the regression grading of Dworak [7,16]. This histological method is based on the stroma/tumor cell relation within the tumor after nCRT. It is a commonly used method to quantify tumor response after neoadjuvant treatment under clinical routine. Unfortunately it is of no value for identification of response to CRT prior to CRT application. Chemoradiation in rectal carcinoma is clinical routine as it is of clinical benefit in certain tumors stages: It is recommended in a neoadjuvant setting prior to surgery in stage UICC II and III rectal carcinomas. It decreases local recurrence rates and may increase anal sphincter preservation [1,2,4,6]. Its effect on survival, especially after good or complete response to nCRT, is currently unclear [1,2,4,6]. However, good or complete response to nCRT is seen in only about 60% of patients [9]. The rest of the patients show moderate or poor response to CRT. In these cases nCRT does not add any benefit to the patients, but increases perioperative risks and postoperative morbidity. Furthermore these patients are unnecessarily exposed to partially severe side effects of CRT [1]. Molecular markers may help to identify responders to nCRT. Tools such as these are urgently needed to predict the risk/benefit ratio for the patients during clinical routine.

## 4. Material and Methods

### 4.1. Patients and Tumor Biopsies

The study was carried out after obtaining ethical approval by the ethical commission University of Erlangen (ID 3784). For the ICPL analysis, 20 patients with histopathology-proven rectal adenocarcinoma (stage UICC II–IV)—which were recruited for a standardized clinical study (CAO/ARO/AIO-04)—were selected after informed consent [12]. Patients with synchronous second colon carcinomas or younger than 18 years of age were excluded. Tumors with a distance of maximal 16 cm from the anal verge (measured by a rigid endoscopy) were classified as rectal carcinomas. Patients received a total dose of 50.4 Gy radiation, fractioned in 28 × 1.8 Gy single applications. During radiation they received either 5-Fluorouracil (5-FU: 1000 mg/m^2^/day; nCRT I) or 5-FU (250 mg/m^2^/day + Oxaliplatin 50 mg/m^2^/day; nCRT II) [12]. Patient characteristics are listed in Table 2.

**Table 2:** Patients and histopathological tumor characteristics of rectal adenocarcinomas which underwent laser capture microdissection and proteome analysis by isotope coded protein label (ICPL); nCRT I: 50.4 Gy + 5-FU; nCRT II: 50.4 Gy + 5-FU/Oxaliplatin.

Patients	nCRT I	nCRT II
n	10	10
Male	9	7
Female	2	3
ypT-category		
yT0	1	3
yT1	0	0
yT2	3	1
yT3	5	6
yT4	1	0
ypN-category		
yN0	4	7
yN1	5	3
yN2	1	0
Distant metastasis		
M0	9	9
M1	1	1
Grading		
G1/2	6	6
G3/4	3	2
GX	1	2
Regression Grading (Dworak)		
Dworak 1	2	0
Dworak 2	3	5
Dworak 3	4	2
Dworak 4	1	3

Tumor biopsies of all patients were performed prior to nCRT by rigid rectoscopy. The samples used for ICPL were immediately harvested in liquid nitrogen and stored by −80 °C until further processing. Samples used for immunohistochemistry examination underwent a formalin fixation and paraffin embedding procedure (FFPE). Standardized surgery such as total mesorectal excision (TME) was performed in all cases.

### 4.2. Study Protocol

**Figure 2:**
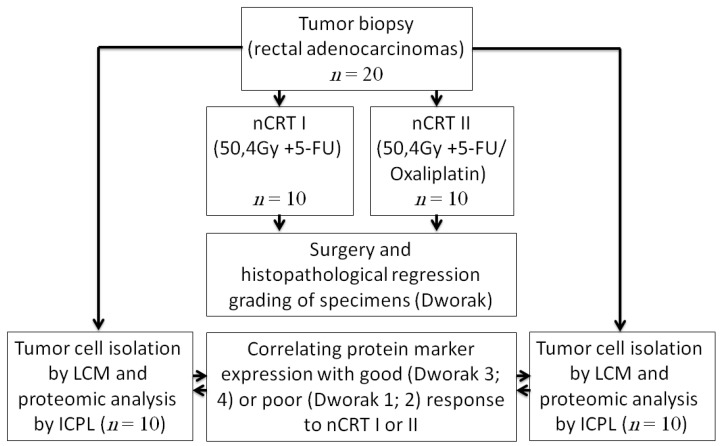
Study protocol; tumors underwent biopsy prior to neoadjuvant chemoradiation (nCRT); nCRT I: 50.4 Gy + 5-FU; nCRT II: 50.4 Gy + 5-FU/Oxaliplatin; tumor cells were isolated by laser capture microdissection (LCM) from the biopsies and proteomic analysis were performed by isotope coded protein label (ICPL). After nCRT and surgery histopathological tumor regression grading was scored (Dworak) and protein marker expression of the tumor biopsies was correlated with specimens’ response to nCRT.

Endoscopic tumor biopsies were obtained from patients with histopathologically confirmed rectal adenocarcinoma prior to nCRT. Six weeks after completion of nCRT, respective surgery was performed and the tumor response to nCRT was classified according to Dworak’s histopathological regression grading. Tumors were separated into two groups regarding the Dworak’s regression grading: Dworak 1 + 2: (poor response), Dworak 3 + 4 (good response) [7,16]. Differences in the tumors’ protein expression were identified by ICPL in the biopsies obtained prior to nCRT, comparing poor (*n* = 10) *vs.* good (*n* = 10) responders (Figure 2).

### 4.3. Laser Capture Microdissection (LCM)

**Figure 3:**
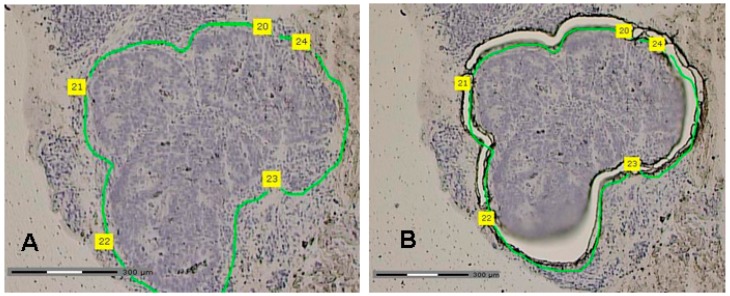
Laser capture micodissection of tumor biopsies. (**A**) The tumor tissue is circled by a green line; (**B**) the laser dissects the tumor tissue which will be picked up and separated for proteomic analysis. Scale bar = 300 µm, numbers are starting points for the laser.

For proteome analysis with ICPL tumor cells were isolated from the biopsies using laser capture microdissection (LCM), according to published protocol [17] (Figure 3). In brief, frozen tissue of each patient was imbedded in OCT tissue freezing medium, cut into 8–10 µm thick sections using Leica Cryostat Jung CM 3000 (Leica Microsystems Vertrieb GmbH, Wetzlar, Germany) at −20 °C, and mounted onto a PET-membrane with 1.0 mm thickness (Carl Zeiss MicroImaging GmbH, Göttingen, Germany). The sectioned tissues were stored for further preparation at −25 °C. For tissue staining, the cresyl violet protocol, Carl Zeiss (Carl Zeiss MicroImaging GmbH, Göttingen, Germany) with minor modification was used. In brief, the sectioned tissues were dried with cold 70% ethanol for 2 min, stained for 30 s in cresyl violet solution containing 1% (*w*/*v*) cresyl violet acetate and 50% ethanol, and rinsed with 70% ethanol followed by 100% ethanol. The stained tissues were air-dried for 2 min and LCM was then performed using PALM MicroBeam MB IV (Carl Zeiss MicroImaging GmbH, Göttingen, Germany) with approximately 100 mm^2^ total area cut per tissue sample, which corresponds to a protein amount of at least 20 µg. Proteins were solubilized out of microdissected tissue for 30 min with 30 µL guanidine/HEPES pH 8.5, centrifuged at 10,000× *g* at 4 °C for 15 min and sonicated 5 times for 10 s. The protein samples were pooled, due to material limitations of biopsies. The pooling was performed in accordance with the histopathological response grading of the specimens to nCRT (Dworak 1 + 2 or Dworak 3 + 4) [7].

### 4.4. Isotope Coded Protein Labeling (ICPL)

Before ICPL-labeling, acetone precipitation of solubilized and pooled proteins from microdissected (LCM) tissue was performed [10,11]. To one volume of each sample 5 volumes of 100% acetone cooled to −20 °C were added and incubated overnight at −20 °C. Precipitated proteins were pelleted by centrifugation 30 min at 20,000× *g* at 4 °C. Pellets were washed twice with cold 80% (*v*/*v*) acetone by centrifugation for 5 min at 20,000× *g* at 4 °C. The protein concentration was determined by a Bradford assay (BioRad Laboratories GmbH, Munich, Germany) before and after acetone precipitation, the last of which was used to adjust sample concentration at 5 µg/µL in ICPL-lysis buffer (40 µL) containing 6 M guanidine-HCL and 0.1 M HEPES pH 8.5. For ICPL-labeling, cysteines were reduced by adding of 1 µL of 2 M TCEP in 0.1 M HEPES pH 8.5 into each of the 4 samples and overfilled with argon. After vortexing for 30 s and 1 min ultrasonic treatment, the samples were incubated for 30 min at 60 °C. For alkylation of cysteines 1 µL of 0.4 M IAA (Iodoacetamide) in 0.1 M HEPES pH8.5 was added into each of the 4 samples and incubated for 30 min at 25 °C. Alkylation was quenched by adding 0.5 M *N*-acetyl cysteine solution in 0.1 M HEPES pH 8.5 followed by incubation for 15 min at 25 °C. For the differential labeling, four ICPL-reagents, ICPL_0_, ICPL_4_, ICPL_6_ and ICPL_10_, were used as a quadruplex approach. Proteins were then labeled with one of the 4 ICPL reagents (6 µL of 0.15 M ICPL_0_, ICPL_4_, ICPL_6_ or ICPL_10_). The reaction was allowed to proceed for 2 h at 25 °C under a nitrogen atmosphere to minimize methionine oxidation. Labeling was quenched by addition of hydroxylamine (1.5 M, pH 8.3), and all four samples were combined and stored by −20 °C. From each of the two separately prepared technical replicates, the sample amount was divided for the OFFGEL-fractionation (95%; 150 µg) and for non-fractionation (5%; 10 µg). For OFFGEL-fractionation , ICPL-labeled and combined proteins (150 µg) were fractionated by isoelectric point (pI) using an Agilent 3100 OFFGEL fractionator (Agilent Technologies, Oberhaching, Germany) according to the manufacturer’s instructions The samples from the first technical replicate (24 OFFGEL-fractions and non-fractionated sample) were analyzed after in-solution digestion with the enzymes Glu-C und Trypsin [17] by the Core Facility (Max-Planck Institute for Biochemistry, Martinsried, Germany) with the LTQ-hybrid mass spectrometer (Thermo Fischer Scientific, Bremen, Germany). Nano-LC-MS/MS was performed on an LTQ-Orbitrap mass spectrometer (Thermo Fisher Scientific, Schwerte) coupled to a nanoHPLC Agilent 1200 system (Agilent Technologies Deutschland GmbH, Böblingen, Germany). Briefly, peptides were preconcentrated on a reversed-phase (RP) trapping column (15 cm-fused silica column with 75 µm ID filled with ReproSil-Pur C18-AQ 3 µm resin (Dr. Maisch GmbH, Ammerbuch-Entringen, Germany) in 0.1% TFA (Trifluoroacetic) using a gradient from 2% to 40% in 0.5% acetic acid in ACN (Acetonitrile) at a flow rate of 250 nL/mL in 100 min.

MS (Mass Spectrometry) survey scans were acquired within the Orbitrap from 300 to 1800 *m*/*z* at a resolution of 60.000. The ten most intense signals were subjected to collision induced dissociation (CID) in the ion trap. The samples from the second technical replicates (24 OFFGEL-fractions and non-fractionated sample) were also analyzed after in-solution digestion with the enzymes Glu-C und Trypsin [18] by the group of A. Sickmann (Leibniz–Institut für Analytische Wissenschaften-ISAS-e.V., Dortmund, Germany) with the LTQ-Orbitrap Velos (Thermo Fisher Scientific, Bremen, Germany). Nano-LC-MS/MS was performed on an LTQ-Orbitrap Velos mass spectrometer (Thermo Fisher Scientific, Bremen, Germany) coupled to an Ultimate 3000 Rapid Separation Liquid Chromatography (RSLC) system (Dionex, Germering, Germany). Briefly, peptides were preconcentrated on a reversed-phase (RP) trapping column (Acclaim PepMap, 75 μm × 2 cm C18, 100 Å, Dionex) in 0.1% TFA followed by RP separation (Acclaim PepMap RSLC 75 μm × 15 cm, 2 μm, 100 Å, Dionex) using a binary gradient (solvent A: 0.1% FA, solvent B: 0.1% FA, 84% ACN) from 5% to 50% B at a flow rate of 300 nL/min in 90 min.

MS survey scans were acquired within the Orbitrap from 300 to 2000 *m*/*z* at a resolution of 60,000 using polysiloxane *m*/*z* 445.120030 as lock mass. The ten most intense signals were subjected to collision induced dissociation (CID) in the ion trap taking into account a dynamic exclusion of 12 s. CID spectra were acquired with a normalized CE (collision energy) of 35%, a default charge state of 2 and an activation time of 30 ms. AGC target values were set to 104 for ion trap MSn and 106 for Orbitrap MS scans.

Since only ICP-samples have been analyzed, these generated MS/MS spectra or MSn spectra were evaluated by the ICPL-ESIQuant software (Max-Planck Institut for Biochemistry, Martinsried, Germany).

### 4.5. Immunohistochemistry

Selection of markers for immunohistochemistry was performed randomly. Four proteins have been selected for immunohistochemical validation in rectal carcinoma biopsies which were sampled prior to nCRT and fixed in formalin and paraffin embedded. Tissue sections (4 µm) were dewaxed in xylene and rehydrated. For staining, antigene retrieval was performed for 20 min at 95 °C in citrate target retrieval solution pH 6.0 (DAKO, Hamburg, Germany) or tissue sections were digested with hyaluronidase from bovine testis (2 mg/mL in PBS, pH 5.5, Sigma Aldrich, Taufkirchen, Germany) and protease from Streptomyces griseus (1 mg/mL in PBS, pH 7.4, Sigma Aldrich) for 30 min each at 37 °C. Sections were incubated with antibodies raised against human anti-HADHA (ab54477, Abcam, Cambridge, UK), anti-SM22 α [1B8] (Transgelin) (ab28811, Abcam, Cambridge, UK), anti-Transketolase [7H1AA1] (ab112997, Abcam, Cambridge, UK) and anti-Plectin [E398P]; (ab32528, Abcam, Cambridge, UK). As a control, sections were incubated with the same concentration of non binding isotypic mouse immunoglobulins. Immunostaining was detected using the ZytoChem-Plus AP (Alkaline phosphatase) polymer kit (Zytomed Systems, Berlin, Germany) and liquid permanent red (DAKO) according to the manufacturers’ instruction. Slides were counterstained with gill-3 haematoxylin (Merck, Darmstadt, Germany), air-dried and mounted with VectaMount permanent mounting medium (Vector Laboratories, Burlingame, CA, USA) (Figure 1).

### 4.6. Statistics

Identification and quantification of the proteins were examined separately for the nCRT I and nCRT II group. Before quantification, the raw files were de-isotoped and deconvoluted by the extract function of the Trans Proteomic Pipeline software (TPP); Version 4.3 (Seattle Proteome Center, Washington, WA, USA) and transformed to an mzXML data format. Then quantification was performed using the ICPL-ESI*Quant* version 2.0 software [19]. Selected parameters were chosen as follows: mass accuracy 60 ppm, co-elution count: ≥2 and ICPL (label 0, monoisotopic mass = 105.02), ICPL:2H(4) (label 4, monoisotopic mass = 109.04), ICPL:13C(6) (label 6, monoisotopic mass = 111.04), and ICPL:13C(6)–2H(4) (label 10, monoisotopic mass = 115.06). For identification, proteins were searched against the IPI (International Protein Index) database using of MASCOT, Version 2.2 (Matrix Science, London, UK) with the following parameter settings: Homo sapiens as organism, carbamidomethylation (C) as a fixed modification, variable modification of oxidized methionine, all 4 variable Protein Nterm and ICPL(K) labels, trypsin digestion with one missed cleavage allowed, minimum Mascot protein score ≥60.0 and minimum Mascot peptide score ≥20.0. For the reliability of the reported database search results an automatic decoy database search was performed with the same selected parameters as described above.

### 4.7. Quantification and Identification of Potential Protein Signatures

The ICPL-ESIQuant software (Max-Planck Institut for Biochemistry, Martinsried, Germany) was used for the evaluation of the total 50 individual samples (2 × 24 OFFGEL-fractions and 2× non-fractionated samples). Proteins are considered to be quantified and identified, if at least two multiplets per protein and a unique peptide per protein were available. In case of a protein that was identified in neighboring OFFGEL-fractions, this protein was regarded as a single protein species. In case of a protein identified in several OFFGEL-fractions, but not in between two fractions, this protein was regarded as a separate protein species or isoform.

The identified proteins were classified as differentially expressed with a regulation value of 1.5 ≥ 1 ≥ 0.66, with at least two quadruplets per protein and a unique peptide per protein (CV ≤ 30%).

## 5. Conclusions

In summary we identified a panel of proteome markers which may act as response predictors for neoadjuvant CRT in rectal carcinomas in the future. The main limitation of our study is a limited patient cohort from which we derived our data. For this reason our results have to be considered as preliminary. In a next step these markers must be validated in an independent patient cohort prospectively to elucidate its value for clinical use.
